# The predictive significance of lipid accumulation products for future diabetes in a non-diabetic population from a gender perspective: an analysis using time-dependent receiver operating characteristics

**DOI:** 10.3389/fendo.2023.1285637

**Published:** 2023-11-14

**Authors:** Jiajun Qiu, Maobin Kuang, Yang Zou, Ruijuan Yang, Qing Shangguan, Dingyang Liu, Guotai Sheng, Wei Wang

**Affiliations:** ^1^Department of Internal Medicine, Medical College of Nanchang University, Jiangxi Provincial People’s Hospital, Nanchang, Jiangxi, China; ^2^Jiangxi Cardiovascular Research Institute, Jiangxi Provincial People’s Hospital, The First Affiliated Hospital of Nanchang Medical College, Nanchang, Jiangxi, China; ^3^Department of Endocrinology, Jiangxi Provincial People’s Hospital, The First Affiliated Hospital of Nanchang Medical College, Nanchang, Jiangxi, China; ^4^Jiangxi Provincial Geriatric Hospital, Jiangxi Provincial People’s Hospital, The First Affiliated Hospital of Nanchang Medical College, Nanchang, Jiangxi, China

**Keywords:** time-dependent ROC analysis, prediction, lipid accumulation products, gender, diabetes, LAP

## Abstract

**Objective:**

The increasing prevalence of diabetes is strongly associated with visceral adipose tissue (VAT), and gender differences in VAT remarkably affect the risk of developing diabetes. This study aimed to assess the predictive significance of lipid accumulation products (LAP) for the future onset of diabetes from a gender perspective.

**Methods:**

A total of 8,430 male and 7,034 female non-diabetic participants in the NAGALA (NAfld in the Gifu Area, Longitudinal Analysis) program were included. The ability of LAP to assess the risk of future new-onset diabetes in both genders was analyzed using multivariate Cox regression. Subgroup analysis was conducted to explore the impact of potential modifiers on the association between LAP and diabetes. Additionally, time-dependent receiver operator characteristics (ROC) curves were used to assess the predictive power of LAP in both genders for new-onset diabetes over the next 2-12 years.

**Results:**

Over an average follow-up of 6.13 years (maximum 13.14 years), 373 participants developed diabetes. Multivariate Cox regression analysis showed a significant gender difference in the association between LAP and future diabetes risk (*P*-interaction<0.05): the risk of diabetes associated with LAP was greater in females than males [hazard ratios (HRs) per standard deviation (SD) increase: male 1.20 (1.10, 1.30) vs female 1.35 (1.11, 1.64)]. Subgroup analysis revealed no significant modifying effect of factors such as age, body mass index (BMI), smoking history, drinking history, exercise habits, and fatty liver on the risk of diabetes associated with LAP (All *P*-interaction <0.05). Time-dependent ROC analysis showed that LAP had greater accuracy in predicting diabetes events occurring within the next 2-12 years in females than males with more consistent predictive thresholds in females.

**Conclusions:**

This study highlighted a significant gender difference in the association between LAP and future diabetes risk. The risk of diabetes associated with LAP was greater in females than in males. Furthermore, LAP showed superior predictive ability for diabetes at different time points in the future in females and had more consistent and stable predictive thresholds in females, particularly in the medium and long term.

## 1 Introduction

Diabetes is a global public health concern, with increasing rates of morbidity and mortality (1). Thankfully, however, early intervention can be effective in delaying and reducing the development of diabetes in high-risk individuals (2–5). To achieve this goal, identifying and detecting individuals at high risk for diabetes early on is essential (6, 7). Previous studies have shown that there were significant gender differences in the prevalence and progression of diabetes (8, 9) and that such differences varied over time (10, 11). Therefore, in the new era of precision medicine, it is imperative to quantify future diabetes risk in both genders, assess trends in at-risk populations, and provide primary care physicians with references for gender-specific interventions, thereby reducing the incidence of diabetes.

Currently, diabetes is in the midst of a rapidly growing pandemic and most arguments support the idea that the growing trend of diabetes is mainly attributable to the obesity epidemic (12, 13). Obesity primarily manifests as the accumulation of subcutaneous adipose tissue (SAT) and VAT (14, 15). However, growing evidence suggested that VAT, an ectopically deposited adipose tissue, exhibits a stronger association with insulin resistance than SAT and plays a more important role in the development of diabetes (16–18). Moreover, gender disparities in visceral adiposity in the microenvironment significantly impact the development of diabetes (19, 20). Therefore, the quantification of VAT may provide a more effective means of assessing diabetes risk in diverse gender populations and accurately predicting its occurrence and future trends. However, precise VAT measurement requires abdominal computed tomography or nuclear magnetic resonance imaging scans that are costly and less accessible (21), hindering their application in large-scale population-based health screenings. Therefore, it becomes imperative to develop alternative approaches for a simple yet effective VAT assessment.

In recent years, the LAP, calculated by Kahn and colleagues using waist circumference (WC) and triglyceride (TG) levels, has emerged as a valid alternative method for assessing VAT (22, 23). Numerous epidemiological studies have independently linked LAP to future diabetes risk (23–35) and demonstrated its predictive value for future diabetes (24–31). A recent meta-analysis further highlighted that LAP exhibited a stronger association with diabetes risk in females and had a superior predictive capability for females compared to males (36). However, these predictive values obtained through traditional ROC analyses only focused on the ability of LAP in predicting new-onset diabetes at a specific time point, and it remains unclear whether LAP’s predictive performance differs between genders over varying future time periods. Notably, males possess more diabetes risk habits and factors (e.g., smoking and drinking, fatty liver, and obesity) than females (8, 37, 38), and hormone levels can also significantly fluctuate between genders over time (10). As a result, the association between baseline LAP and diabetes risk, as well as LAP’s predictive efficacy for new-onset diabetes, may change over time due to these factors and others. Therefore, it is imperative to quantify the time-varying predictive ability of LAP for future new-onset diabetes in both males and females. This approach can assist clinicians in offering personalized intervention recommendations based on gender and time considerations for individuals at risk of diabetes. Additionally, it provides governments with valuable insights to develop targeted diabetes prevention strategies tailored to specific populations (9). To address the aforementioned issues, we assessed the predictive value of LAP for future new-onset diabetes at various time points in a non-diabetic population cohort with a maximum follow-up period of 13 years and explored gender disparities.

## 2 Materials and methods

### 2.1 Data source

This study was a post hoc analysis of a large longitudinal cohort of the NAGALA project. Professor Okamura has uploaded data from the NAGALA project to the Dryad database (39), which we used for analysis without infringing on the rights of the data providers to longitudinally assess gender differences in the predictive value of LAP for future diabetes. Our study may prompt researchers to include gender considerations in diabetes prevention studies and also inform the development of gender-specific diabetes precision prevention strategies. The Ethics Committee of Jiangxi Provincial People’s Hospital approved the current research protocol (Institutional Review Board Study Number:2021-066); In addition, according to local laws and regulations, the Ethics Committee of Jiangxi Provincial People’s Hospital exempted the repeated signing of informed consent of subjects.

**Figure 1:**
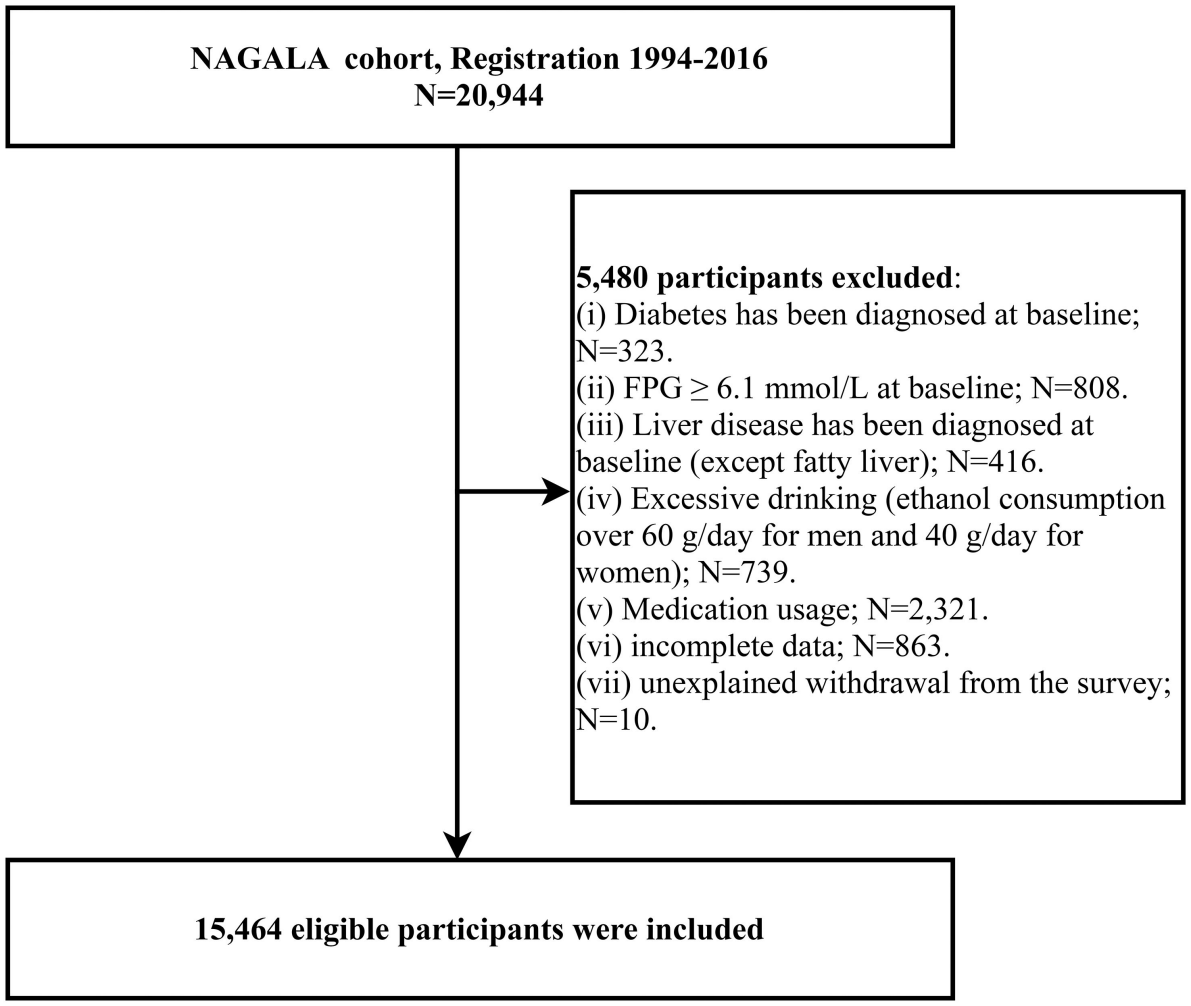
Flowchart of the selection process of study subjects.

A previous study was conducted by Okamura et al. who analyzed the relationship between ectopic fat obesity and diabetes, and the detailed study design and methodology have been previously described (40). Briefly, the purpose of the NAGALA study was to assess chronic diseases and their risk factors, and the original cohort consisted of 20,944 members of the general population who underwent health checkups at Murakami Memorial Hospital in Gifu, Japan, between 1994 and 2016; all participants underwent questionnaires, general body measurements, laboratory blood tests, and liver ultrasound examinations, and data regarding demographic information, lifestyle, personal medical history (disease and medication history), anthropometric parameters, and laboratory indicators were recorded. Based on the purpose of the study, we extracted all the data uploaded by Prof. Okamura in the Dryad database (https://doi.org/10.5061/dryad.8q0p192) and recalculated the gender-specific LAP. Participants with diabetes at baseline (n=323), fasting plasma glucose (FPG) over 6.1 mmol/L at baseline (n=808), liver disease other than fatty liver (n=416), excessive alcohol consumption (over 60 g/day for males and 40 g/day for females) (n=739), medication use at baseline (n=2,321), missing data (n=874), and unexplained withdrawal from the study (n=10) were excluded, and we finally included a total of 15,464 eligible participants. **Figure 1** shows the flow chart of the study population inclusion.

### 2.2 Data acquisition and collection

As described in a previous study (40), information on socio-demographic characteristics, disease history, drug use, smoking status, drinking status, and exercise habits was obtained through a standardized questionnaire; general physical measurements such as height, weight, WC, and blood pressure [systolic blood pressure (SBP), diastolic blood pressure (DBP)] were obtained by trained professionals using standard methods; participants had their venous blood samples collected by experienced medical staff after an overnight fast of 8 hours, and then laboratory parameters including blood glucose [hemoglobin A1c (HbA1c), FPG], lipids [high-density lipoprotein cholesterol (HDL-C), total cholesterol (TC), TG], and liver function enzymes [alanine aminotransferase (ALT), aspartate aminotransferase (AST), gamma-glutamyl transferase (GGT)] were measured using an automated biochemical analyzer. Fatty liver was determined by abdominal ultrasound and assessed by experienced gastroenterologists with unknown patient information based on four known criteria of abdominal ultrasound such as deep attenuation, hepatorenal echo contrast, liver brightness, and vascular blurring (41). Based on regular weekly exercise, study participants were categorized as having and not having exercise habits (42). Drinking status was divided into four categories based on the subject’s weekly alcohol intake during the previous month: Non/small drinking (<40 g/week); light drinking (40-140 g/week); moderate drinking (140-280 g/week); heavy drinking (>280 g/week) (43). Smoking status was divided into three groups based on participants’ tobacco use prior to the baseline survey: non, past, and current.

### 2.3 Calculation of LAP

Males: LAP= [(WC (cm)-65) × TG (mmol/l)];

Females: LAP= [(WC (cm)-58) × TG (mmol/l)] (22).

### 2.4 Prespecified subgroups

BMI subgroups: According to the World Health Organization’s recommended criteria for classifying overweight/obese (BMI ≥ 25 kg/m^2^) and non-obese (BMI <25 kg/m^2^) populations in Asia (44), we defined BMI subgroups using 25 kg/m^2^ as the cut-off.

Age subgroups: Given that the average natural age of menopause in Asian females is around 50 years (45) and that changes in hormone levels during menopause may have an impact on visceral adiposity (46); furthermore, the prevalence of metabolic syndrome in males and females reverses at age 50 years (47). Therefore, we used 50 years as a cut-off to define age subgroups.

Smoking history subgroups: Smoking history subgroups were redefined according to smoking status at baseline, with non-smokers defined as having no smoking history and with past or current smokers defined as having a smoking history.

Drinking history subgroups: The drinking history subgroup was redefined based on drinking status at baseline, with those who did not drink or rarely drank defined as having no drinking history, and those who drank little amounts, moderate amounts, and large amounts defined as having a drinking history.

Exercise habits subgroups: defined as with and without exercise habits based on the previous grouping of exercise habits.

Fatty liver subgroups: defined as having a fatty liver and not having a fatty liver based on whether or not there was a prior diagnosis of fatty liver.

### 2.5 Diabetes definition

Follow-up began at the time of participants’ baseline recording, and new-onset diabetes was determined according to American Diabetes Association criteria (48). The criteria for diabetes diagnosis were (1) FPG ≥7.0 mmol/L; (2) HbA1c ≥6.5%; or (3) self-reported diabetes (confirmed by medical personnel).

### 2.6 Statistical analysis

We performed all analyses in this study using R language version 4.2.1 and Empower(R) version 2.20. First, we grouped participants by gender and compared differences in baseline data; in addition, we further compared differences in baseline data between participants with and without future diabetes in both genders. Weighted standardized differences were calculated by the inverse probability of treatment weighting method to quantify and assess between-group differences, where standardized differences >10% were considered to be significantly different (49).

Multivariate Cox proportional hazards regression models were constructed to explore the relationship between LAP and diabetes risk, and HRs were calculated to quantify the risk of new-onset diabetes associated with each SD increase in LAP in both genders. To fully consider the effect of covariates on the outcome, we identified confounders to be adjusted from data including demographic information, body measurement parameters, lifestyle, and laboratory indicators by covariance diagnosis, where covariates with a final variance inflation factor less than 5 were included in the model for adjustment (50), and **Supplementary Table 1** provides more detailed information on the selection of covariates; in addition, the proportional hazards assumption was confirmed in the current study by constructing Kaplan-Miere curves for the LAP quartiles. To control for confounding factors that may affect the outcome, we constructed four stepwise adjusted Cox regression models in the whole population and in both genders, respectively. Specifically, Model 1 was an age-adjusted model; Model 2 was further adjusted for height, fatty liver, exercise habits, drinking status, and smoking status; Model 3 was further adjusted for liver function enzymes such as ALT, AST, GGT; Model 4 was used as the final model and adjusted for all non-colinear variables. In addition, we performed an interaction test based on Model 4 to assess for differences in LAP-related diabetes risk in both genders.

To account for the influence of potential effect modifiers on the results, we further performed subgroup analyses in both genders, prespecifying some subgroups, including age, BMI, smoking history, drinking history, exercise habits, and fatty liver. In the final model, interaction tests were performed using likelihood ratio tests to evaluate for differences in the risk of diabetes associated with LAP across subgroups. Finally, we also used time-dependent ROC curves to evaluate the predictive ability and stability of LAP for new-onset diabetes in different periods in the next 2-12 years in both genders and recorded the corresponding prediction thresholds, sensitivities, and specificities, and the area under the curve (AUC) at different follow-up time nodes.

## 3 Results

### 3.1 Baseline characteristics of the study population

The original cohort comprised a total of 20,944 individuals, and 15,464 participants were included after applying screening criteria. Of these, 8,430 (54.5%) were male and 7,034 (45.5%) were female. As expected, baseline data for male and female participants exhibited significant differences (**Table 1**). Males had greater values for height, weight, BMI, WC, SBP, DBP, FPG, liver function enzymes (ALT, AST, GGT), TG, and LAP over females. Additionally, a higher proportion of males had a history of smoking, drinking, fatty liver, and diabetes. In contrast, the HDL-C level was significantly higher in female participants than in males.

**Table 1:** Baseline demographic, lifestyle, and laboratory characteristics in participants classified by sex.

	Female	Male	Standardized Difference (95% CI), %
Participants(n)	7034	8430	
Diabetes	87 (1.24%)	286 (3.39%)	14 (11, 18)
Age(years)	43.25 (8.76)	44.09(9.00)	9 (6, 13)
Height(m)	1.58 (0.05)	1.71 (0.06)	218 (214, 222)
Weight(kg)	52.66 (7.87)	67.29 (9.93)	163 (160, 167)
BMI (kg/m^2^)	21.01(2.93)	23.04 (2.98)	69 (66, 70)
WC (cm)	71.68 (8.08)	80.47 (7.91)	110 (107, 113)
ALT (IU/L)	14.00 (11.00-17.00)	20.00 (16.00-28.00)	67 (64, 70)
AST (IU/L)	16.00 (13.00-19.00)	18.00 (15.00-23.00)	36 (33, 39)
GGT (IU/L)	12.00 (10.00-15.00)	20.00 (15.00-29.00)	74 (71, 77)
HDL-C (mmol/L)	1.65 (0.38)	1.31 (0.35)	94 (90, 97)
TC (mmol/L)	5.09 (0.88)	5.16 (0.85)	9 (5, 12)
TG (mmol/L)	0.56 (0.41-0.81)	0.93 (0.63-1.39)	75 (72, 79)
HbA1c (%)	5.18 (0.32)	5.16 (0.32)	7 (3, 10)
FPG (mmol/L)	4.99 (0.39)	5.31 (0.37)	84 (81, 88)
SBP (mmHg)	109.36 (14.32)	118.78 (14.14)	66 (63, 69)
DBP (mmHg)	67.64 (9.76)	74.87 (9.96)	73 (70, 77)
Fatty liver	486 (6.91%)	2255 (26.75%)	55 (52, 58)
Exercise habits	1109 (15.77%)	1600 (18.98%)	8 (5, 12)
LAP	6.65 (3.48-12.59)	13.63 (6.93-25.40)	59 (55, 62)
Drinking status			74 (71, 77)
Non/small	6451 (91.71%)	5354 (63.51%)	
Light	389 (5.53%)	1369 (16.24%)	
Moderate	194 (2.76%)	1166 (13.83%)	
Heavy	0 (0.00%)	541 (6.42%)	
Smoking status			129 (126, 133)
None	6139 (87.28%)	2892 (34.31%)	
Past	441 (6.27%)	2511 (29.79%)	
Current	454 (6.45%)	3027 (35.91%)	

Values were expressed as mean (SD) or medians (quartile interval) or n (%). Abbreviations, BMI, body mass index; WC, waist circumference; ALT, alanine aminotransferase; AST, aspartate aminotransferase; GGT, gamma-glutamyl transferase; HDL-C, high-density lipoprotein cholesterol; TC, total cholesterol; TG, triglyceride; HbA1c, hemoglobin A1c; FPG, fasting plasma glucose; SBP, systolic blood pressure; DBP, diastolic blood pressure; LAP, lipid accumulation product.

We also summarized the clinical characteristics of male and female participants at baseline according to their future occurrence or absence of diabetes (**Table 2**). Significant differences were already observed between the diabetic and non-diabetic groups at baseline in both genders. In females, the diabetic group had higher values for age, weight, BMI, WC, ALT, AST, GGT, TC, TG, HbA1c, FPG, SBP, DBP, LAP, rates of fatty liver disease, and alcohol consumption, whereas the non-diabetic group exhibited greater height and HDL-C; exercise habits and smoking status did not show significant differences between the two groups. In males, however, all covariates exhibited significant differences between the diabetic and non-diabetic groups at baseline (all standardized differences >10%). The diabetic group had a higher rate of smoking, while the non-diabetic group had a higher rate of exercise. Other baseline characteristics showed similar differences as in the female group.

**Table 2:** Baseline demographic, lifestyle, and laboratory characteristics in participants classified by the presence of different sex and incidence of diabetes.

	Female			Male		
	Non-diabetic	Diabetic	Standardize Difference (95% CI), %	Non-diabetic	Diabetic	Standardize Difference (95% CI), %
Participants(n)	6947	87		8144	286	
Age(years)	43.20 (8.75)	47.60 (8.54)	51 (30, 72)	43.99 (9.00)	47.00 (8.52)	34 (23, 46)
Height(m)	1.58 (0.05)	1.56 (0.06)	41 (19, 62)	1.71 (0.06)	1.70 (0.06)	15 (3, 26)
Weight(kg)	52.58 (7.78)	59.45 (11.09)	72 (51, 93)	67.09 (9.78)	73.00 (12.32)	53 (41, 65)
BMI (kg/m^2^)	20.96 (2.89)	24.47 (4.35)	95 (74, 116)	22.97 (2.92)	25.21 (3.63)	68 (56, 80)
WC (cm)	71.57 (7.96)	80.40 (11.80)	88 (67, 109)	80.26 (7.77)	86.50 (9.22)	73 (61, 85)
ALT (IU/L)	14.00 (11.00-17.00)	19.00 (14.00-23.00)	56 (35, 77)	20.00 (15.00-27.00)	28.00 (20.00-42.75)	58 (46, 70)
AST (IU/L)	16.00 (13.00-19.00)	18.00 (15.00-22.00)	39 (18, 60)	18.00 (15.00-23.00)	26.00 (19.00-39.75)	38 (26, 49)
GGT (IU/L)	12.00 (10.00-15.00)	15.00 (12.00-22.50)	55 (34, 76)	19.00 (15.00-28.00)	26.00 (19.00-39.75)	34 (23, 46)
HDL-C(mmol/L)	1.65 (0.38)	1.39 (0.34)	71 (49, 92)	1.31 (0.35)	1.12 (0.30)	58 (46, 70)
TC (mmol/L)	5.08 (0.87)	5.55 (0.93)	52 (31, 74)	5.15 (0.85)	5.39 (0.89)	28 (16, 40)
TG (mmol/L)	0.56 (0.41-0.80)	0.96 (0.73-1.30)	81 (60, 103)	0.91 (0.63-1.37)	1.42 (0.91-2.13)	58 (46, 70)
HbA1c (%)	5.18 (0.32)	5.58 (0.37)	116 (95, 137)	5.15 (0.31)	5.51 (0.36)	108 (96, 120)
FPG (mmol/L)	4.98 (0.39)	5.47 (0.41)	123 (101, 144)	5.29 (0.37)	5.66 (0.33)	105 (93, 117)
SBP (mmHg)	109.27 (14.28)	117.05 (15.04)	53 (32, 74)	118.62 (14.06)	123.54 (15.46)	33 (22, 45)
DBP (mmHg)	67.58 (9.75)	72.86 (9.27)	56 (34, 77)	74.74 (9.93)	78.49 (10.16)	37 (26, 49)
Fatty liver	445 (6.41%)	41 (47.13%)	104(82, 125)	2073 (25.45%)	182 (63.64%)	83(71, 95)
Exercise habits	1097 (15.79%)	12 (13.79%)	6 (-16, 27)	1561 (19.17%)	39 (13.64%)	15 (3, 27)
LAP	6.60 (3.45-12.42)	19.74 (12.64-34.99)	91 (70, 112)	6.60 (3.45-12.42)	19.74 (12.64-34.99)	91 (70, 112)
Drinking status			17 (-4,38)			18 (6,29)
Non/small	6369 (91.68%)	82 (94.25%)		5170 (63.48%)	184 (64.34%)	
Light	387 (5.57%)	2 (2.30%)		1331 (16.34%)	38 (13.29%)	
Moderate	191 (2.75%)	3 (3.45%)		1132 (13.90%)	34 (11.89%)	
Heavy	0 (0.00%)	0 (0.00%)		511 (6.27%)	30 (10.49%)	
Smoking status			25 (4, 46)			27 (15, 39)
None	6069 (87.36%)	70 (80.46%)		2817 (34.59%)	75 (26.22%)	
Past	436 (6.28%)	5 (5.75%)		2439 (29.95%)	72 (25.17%)	
Current	442 (6.36%)	12 (13.79%)		2888 (35.46%)	139 (48.60%)	

Values were expressed as mean (SD) or medians (quartile interval) or n (%). All abbreviations as in **Table ​1**.

### 3.2 Association of LAP with diabetes risk

We calculated HRs using Cox proportional hazards regression analysis to assess the association between LAP and diabetes risk. The proportional hazard assumption was verified using the Kaplan-Meier method prior to constructing the regression models (**Supplementary Figure 1**). We found no evidence of a violation of the proportional hazard assumption. In addition, we Z-transformed the independent variable LAP before incorporating it into the Cox regression models to assess the impact of each SD increase in LAP on the risk of diabetes. Finally, four multivariate regression models were constructed for the entire population and for each gender subgroup, and the main association analysis results were presented in **Table 3**. Overall, LAP was independently positively associated with future diabetes risk in the whole population and in gender subgroups [Mode 4: HR per SD increase: total population 1.22 (1.13, 1.32); female 1.35 (1.11, 1.64); male 1.20 (1.10, 1.30)]. The results of gender grouping showed that in all models, the risk of diabetes associated with each SD increase of LAP in females was higher than that in males [HR per SD increase: Mode 1: female 1.97 (1.78, 2.18) VS male 1.48 (1.40, 1.56); Mode 2: female 1.55 (1.34, 1.78) VS male 1.31 (1.23, 1.40); Mode 3: female 1.53 (1.33, 1.77) VS male 1.27 (1.18, 1.36); Mode 4: female 1.35 (1.11, 1.64) vs male 1.20 (1.10, 1.30)]. Furthermore, an interaction test in Model 4 indicated a significant difference in LAP-related diabetes risk between genders (*P* for interaction <0.05).

**Table 3:** Cox regression analyses for the association between LAP and incident DM in different models grouped by sex.

	Hazard ratios (95% confidence interval)				
	Model 1	Model 2	Model 3	Model 4	*P* for interaction
**Total**	1.53 (1.46, 1.61)	1.35 (1.27, 1.43)	1.31 (1.23, 1.40)	1.22 (1.13, 1.32)	
**Sex**					0.03
Female (per SD increase)	1.97 (1.78, 2.18)	1.55 (1.34, 1.78)	1.53 (1.33, 1.77)	1.35 (1.11, 1.64)	
Male (per SD increase)	1.48 (1.40, 1.56)	1.31 (1.23, 1.40)	1.27 (1.18, 1.36)	1.20 (1.10, 1.30)	

LAP, metabolic score for insulin resistance.

Model 2 adjusted for age.

Model 2 adjusted for age, height, fatty liver, exercise habits, drinking status and smoking status.

Model 3 adjusted for age, height, fatty liver, exercise habits, drinking status. smoking status, ALT, AST and GGT.

Model 4 adjusted for age, height, fatty liver, exercise habits, drinking status, smoking status, ALT, AST, GGT, TC, HDL-C, HbA1c and SBP.

### 3.3 Subgroup analysis of gender differences

**Figure 2:**
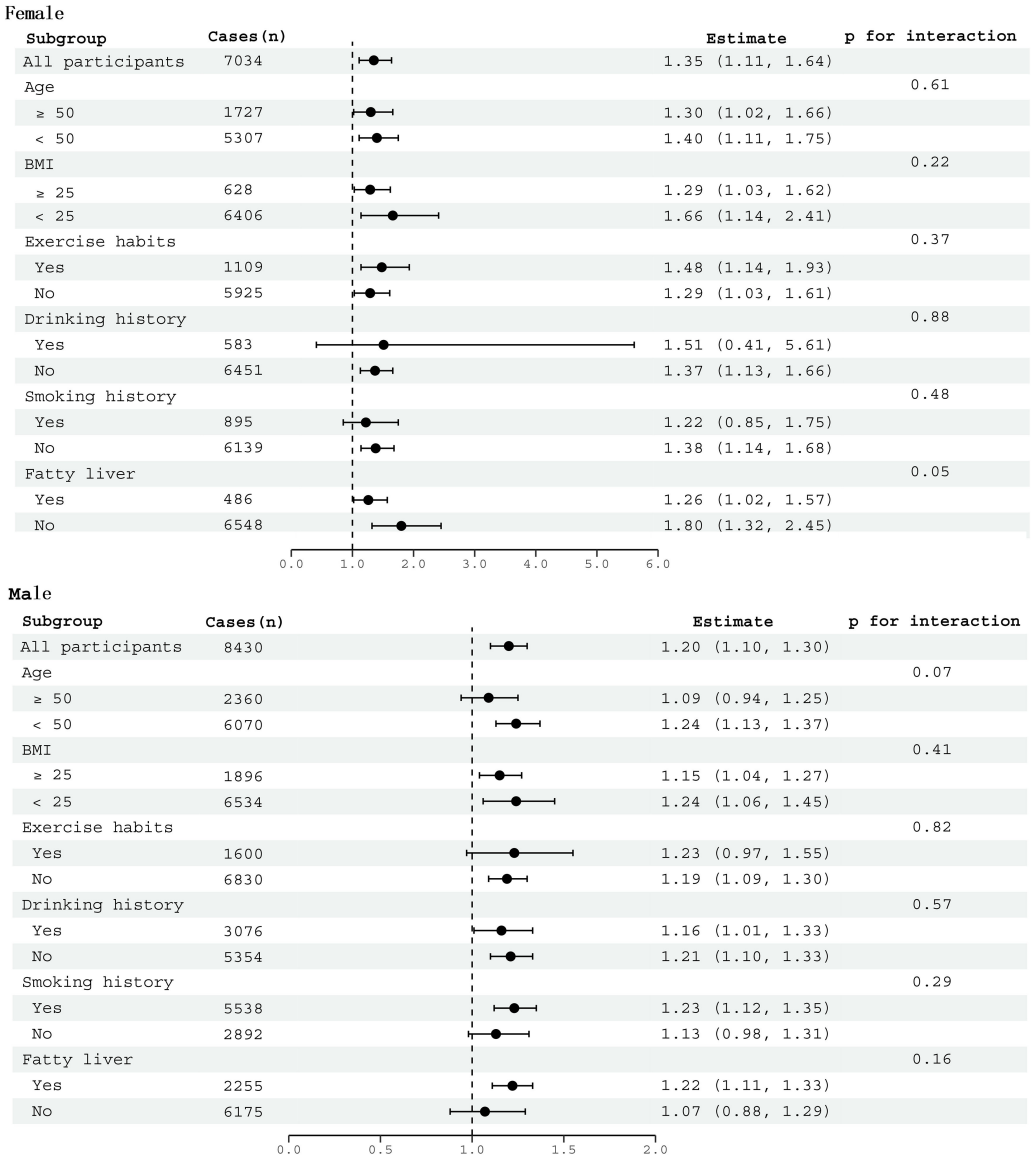
Subgroup analysis between LAP and diabetes by age, BMI, smoking history, drinking history, exercise habits, and fatty liver in males and females.

We conducted subgroup analyses individually for males and females to investigate the relationship between LAP and diabetes risk within specific subgroups. As shown in **Figure 2**, factors such as age, BMI, smoking status, drinking status, exercise habits, and fatty liver had no significant impact on the association between LAP and diabetes risk in both genders (All *P* for interaction >0.05).

### 3.4 Predictive value of LAP for the occurrence of diabetes in both genders

**Figure 3:**
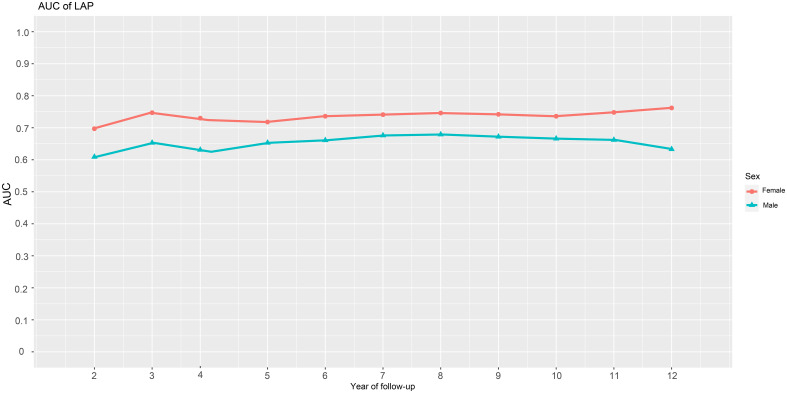
AUC fluctuations of the LAP for predicting the onset of diabetes in males and females. AUC, area under the curves; LAP, lipid accumulation products.

We generated time-dependent ROC curves to assess the predictive accuracy of LAP for new-onset diabetes in males and females at various time points in the future. **Table 4** shows the prediction threshold, sensitivity, specificity, and AUC of LAP for the occurrence of diabetes at 11-time points over 2-12 years in the future in both genders. **Figure 3** shows the trend of the AUC of LAP by gender over time. In general, LAP demonstrated greater accuracy in predicting future diabetes in females than males over the next 2-12 years [AUC: year-2 (female) 0.70 >0.61 (male); year-3 (female) 0.75 >0.65(male); year-4: (female) 0.72 >0.62 (male); year-5 (female) 0.72 >0.65 (male); year-6: (female) 0.74 >0.66 (male); year-7: (female) 0.74 >0.68 (male); year-8 (female) 0.75 >0.68 (male); year-9 (female) 0.74 >0.67 (male); year-10 (female) 0.74 >0.67 (male); year-11 (female) 0.76 >0.66 (male); year-12 (female) 0.76 >0.63 (male)]. Through further longitudinal analysis, we observed that LAP maintained good predictive value for short-term diabetes risk (2-5 years) in females, with AUC values being consistently above 0.7 (2-5 years AUCs were 0.70, 0.75, 0.72, and 0.72, respectively). LAP also demonstrated high predictive accuracy and stability for medium to long-term diabetes risk (6-12 years) in females, with all AUCs above 0.74. Conversely, in males, the predictive value of LAP fluctuated in the short-term (2-6 years) and gradually decreased in the long-term follow-up (8-12 years), with AUCs of 0.68, 0.67, 0.67, and 0.63 for years 8-12, respectively.

**Table 4:** Best threshold, sensitivity, specificity and areas under the time-dependent receiver operating characteristic curves for LAP predicting future diabetes risk for women and man.

	Female				Male			
	Best threshold	Sensitivity	Specificity	AUC	Best threshold	Sensitivity	Specificity	AUC
2-years	8.91	0.80	0.62	0.70	26.02	0.46	0.76	0.61
3-years	12.62	0.73	0.75	0.75	24.41	0.54	0.74	0.65
4-years	10.94	0.74	0.70	0.72	25.20	0.49	0.75	0.62
5-years	12.62	0.65	0.75	0.72	22.13	0.56	0.70	0.65
6-years	11.23	0.71	0.71	0.74	16.62	0.68	0.59	0.66
7-years	11.23	0.72	0.69	0.74	16.94	0.69	0.60	0.68
8-years	11.65	0.70	0.72	0.75	22.29	0.58	0.71	0.68
9-years	7.90	0.82	0.58	0.74	22.35	0.58	0.71	0.67
10-years	12.74	0.64	0.76	0.74	17.78	0.67	0.62	0.67
11-years	11.35	0.73	0.72	0.75	17.79	0.65	0.62	0.66
12-years	11.19	0.76	0.72	0.76	18.55	0.59	0.64	0.63

AUC, area under the curve; other abbreviations as in **Table ​1**.

### 3.5 The threshold value analysis of LAP for prediction of future diabetes in both genders

**Figure 4:**
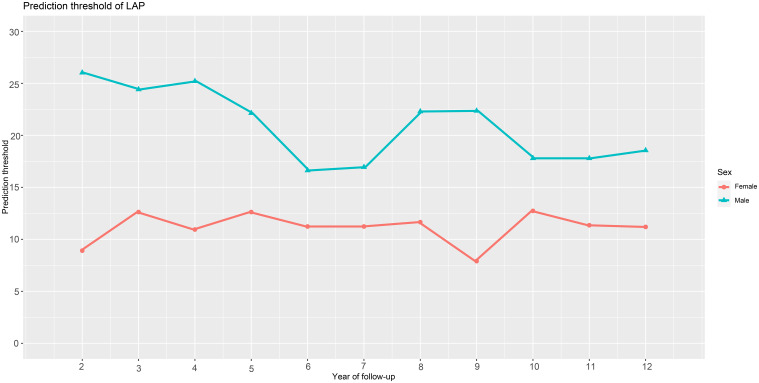
Threshold fluctuations of the LAP for predicting the onset of diabetes in males and females. LAP, lipid accumulation products.

We utilized time-dependent ROC curves to determine the predictive thresholds for detecting future diabetes occurrence at various time points. Detailed information was presented in **Table 4**, and the trend of predictive thresholds for LAP in males and females over time was illustrated in **Figure 4**. Notably, males consistently exhibited higher prediction thresholds compared to females across all 11-time points spanning 2-12 years. Moreover, we observed that the predictive thresholds of LAP showed larger fluctuations in males (predictive threshold range in males: 16.62-26.02) than in females (predictive threshold range in females: 7.90-12.74).

## 4 Discussion

The current study evaluated the predictive value of LAP for future new-onset diabetes at different times in both genders using physical examination data from 15,464 participants in the NAGALA longitudinal cohort. Based on standardized HRs calculated from multivariate Cox regression models, we found that LAP was independently associated with future diabetes risk in the whole population, and after further differentiation by gender, the results showed that an increase in LAP per SD had a greater effect on diabetes risk in females than in males. In addition, subgroup analyses suggested that age, BMI, smoking history, drinking history, exercise habits, and fatty liver did not significantly modify the association between LAP and diabetes risk in both genders. As a primary finding, we also found that the predictive value and stability of LAP for future new-onset diabetes was higher in females than in males over the next 2-12 year period.

### 4.1 Compare the differences between new results and old literature

Diabetes has emerged as a leading cause of mortality and economic burden worldwide (1). Fortunately, effective prevention strategies can avoid or delay the onset of diabetes. Early identification of high-risk individuals and adoption of population-specific targeted preventive measures are the core of precise prevention of diabetes (51). LAP, a simple parameter, has gained extensive attention in recent years for assessing visceral fat. This parameter was developed by Kahn and colleagues (22), who first discovered and identified its potential application in diabetes identification (23). Subsequently, more studies have explored the relationship between LAP and diabetes. Overall, there is a positive correlation between LAP and diabetes risk (24–35, 52–61), and LAP has been found to be effective in diagnosing/predicting diabetes (24–31, 56–62). However, further differentiation between genders in the studies revealed some interesting trends, with two studies (26, 27) showing stronger associations of LAP with diabetes in males than in females; furthermore, in a study involving the general Indonesian population (63), the cross-sectional analysis showed that LAP was associated with diabetes risk in females, while this association disappeared in the male population, and in a subsequent study of 2,427 baseline non-diabetic participants with 4-year follow-up period, LAP was not associated with the risk of future diabetes in either gender. In another cross-sectional study of 430 Brazilian hypertensive people (64), Marcadenti A et al. showed that LAP was positively correlated with the incidence of diabetes in females, while there was no significant association in the male population. Nevertheless, most studies (28–34, 54–57) still supported a stronger association of LAP with diabetes in females than in males, which is consistent with the conclusions proposed in a recent meta-analysis (36) conducted by Khanmohammadi S et al. Our results in a cohort with up to 13 years of follow-up supported this conclusion, and in addition, we did not find effect modifiers that significantly affected the relationship between LAP and diabetes in either gender in the subgroup analysis, and the findings provided new evidence for the previous conclusion. Together, these findings underscored the notion that greater attention needs to be paid to the impact of gender differences when assessing the relationship between visceral fat and future diabetes risk.

Several prior prospective studies have explored the predictive value of LAP for future diabetes (24–31), but only a few have specifically compared its accuracy in male and female subgroups. Among them, Bozorgmanesh M et al. conducted a retrospective study using data from the Tehran Lipid and Glucose Study (29). They assessed the predictive value of LAP for future new-onset diabetes in different populations based on age and gender groupings and then showed that LAP was more accurate in predicting future diabetes in females in the 20-49-year age group and in males in the >50-year age group. In contrast, three cohort studies conducted in Taiwan, China (26), Korea (27), and rural China (28) all showed higher predictive accuracy of LAP for diabetes in females than in males. It is important to note that under natural conditions, outcome events will change over time, and the traditional ROC analysis previously used to evaluate the predictive value of LAP only considered the final binary classification results and ignored the impact of time (26–29). In view of this, in the current study, on the basis of the previous study, we further included the time factor in the ROC analysis for evaluation, and tested the predictive value of LAP for new-onset diabetes in the next 2-12 years by constructing ROC curves of multiple time points. Our new results, after accounting for time factor in the ROC analysis, further demonstrated that LAP predicted diabetes more accurately in females than in males over a period of 2 to 12 years. In addition, our study further showed that LAP also had high prediction stability in predicting the occurrence of diabetes at different time points in the future.

Predictive thresholds for LAP to predict diabetes in both genders have also been previously reported in three studies, including populations in Taiwan (26), Korea (27), and rural China (28). In summary, there was some variation in LAP predictive thresholds for different regional populations, and in addition, the results of studies regarding LAP predictive thresholds in different genders were also inconsistent. Among them, in a large prospective study involving a Taiwanese population in China (26), Chung et al. evaluated the value of different parameters for predicting future diabetes, and the ROC analysis showed that the threshold for LAP to predict diabetes was higher in the male population than in the female. In contrast, in two other prospective studies conducted in Korea (28) and rural China (29), ROC analyses showed that the predictive threshold for LAP to predict diabetes was higher in the female population. In this current longitudinal study, taking into account the time factor in the ROC analysis, we recorded predictive thresholds for LAP to predict the onset of diabetes over the next 2-12 years. The new results showed that the thresholds of LAP in males were consistently higher than that of in females at different time points in the next 2-12 years, which is similar to the report of Chung et al. (26). In addition, it should be noted that, based on different time points in our study, the fluctuation of LAP prediction thresholds for future diabetes was smaller in females, while it was greater in males. Therefore, we concluded that LAP was a more appropriate parameter for predicting future diabetes in females.

Several plausible explanations account for the gender differences observed in the association between LAP and diabetes. First, the composition of human adipose tissue is sexually dimorphic (65) and a study by Machann J et al. (66) showed that females had higher subcutaneous fat and lower visceral fat compared to males; in another study, Bouchard C et al. reported (67) that males had significantly higher levels of VAT compared to females, both in obese and non-obese populations. Additionally, Sex hormone effects also contribute to differences in fat distribution, with estrogen promoting SAT accumulation (20) and testosterone favoring visceral fat accumulation (68); these differences may underlie the gender differences in diabetes risk. In summary, females generally have a lower propensity to accumulate visceral fat compared to males. Consequently, if females were to gain the same amount of visceral fat as males, it could potentially indicate an elevated risk of developing diabetes. On the other hand, a study by Kim EH et al. (69) suggested that as visceral fat area increased from the first quartile to the fourth quartile, the risk of developing diabetes increased from 5 times to 10 times in females than in males, i.e., as visceral fat increased, females were more susceptible to diabetes than males. Therefore, visceral fat represented by LAP in the female population may be more sensitive for predicting diabetes (20) and may have higher predictive accuracy. The detailed mechanisms are not known and may be related to differences in the effects of sex hormones on the storage and metabolism of adipose tissue.

### 4.2 Outlook and suggestions for the future

Although there have been numerous studies showing significant gender differences in the onset and progression of diabetes, the current evidence on gender differences has not been assimilated by most healthcare providers and the impact of gender on diabetes has consistently been underestimated in medical practice (9). In the era of precision medicine, there is an urgent need to integrate the influence of gender on diabetes into contemporary medical research and practice, and research based on inherent differences between males and females can inform the promotion of gender-related decision-making in health. In the current study, by constructing ROC curves of LAP to predict diabetes events at different future time points for both sexes, we confirmed that, in terms of diabetes prediction, LAP was a more suitable obesity parameter for females and had a high predictive value. On the other hand, we also found that the risk of diabetes associated with each SD increase in LAP was higher in females than in males, suggesting that the risk of diabetes was higher in females than in males when LAP increases by the same amount, therefore, females should pay more attention to the increase in visceral fat represented by LAP, and timely control of visceral fat can effectively reduce the incidence of diabetes. Moreover, evidence from intervention studies showed that (70) weight loss strategies including exercise preferentially reduced visceral fat rather than subcutaneous fat, which was more conducive to reducing the risk of diabetes caused by obesity. Together, these results suggested that the female population may benefit more from weight control than the male population in preventing diabetes. Based on these findings, conducting a single health screening that includes assessing LAP levels is also of great significant for predicting the future onset of diabetes at different stages in females. To some extent, this can not only reduce the socioeconomic burden related to diabetes, but also reduce national medical expenditure and resource investment in diabetes prevention, especially in low-income countries.

### 4.3 Strengths and limitations

There are some limitations to this study that should not be overlooked: Firstly, as the baseline data were collected prior to the diagnosis of diabetes, any incorrect recall could have attenuated the true association. Second, 2-hour postprandial glucose was not evaluated in this study, and therefore the possibility of underestimating the incidence of diabetes and resulting in a diminished risk estimate cannot be ruled out. On the other hand, however, although it is possible to underestimate the number of people with diabetes, the results we obtained in a population with a lower incidence than the actual one may be more reliable for the conclusions of the current study. Third, the current cohort study included only the Japanese population, and although the biological mechanisms influencing the development of diabetes in both genders are unlikely to differ significantly compared with populations of other races, and future studies should include the role of race or ethnicity, which the current study population has not been able to assess further. Fourth, nutritional factors are important covariates influencing diabetes risk and it would indeed be meaningful to further consider nutritional factors in the model. However, unfortunately, since our current study is a secondary analysis of an existing dataset, the dataset is fixed and cannot be further updated. Therefore, in the present study, we were unable to obtain sufficient nutritional information for further modeling analysis. It is hoped that further research will incorporate nutritional factors for consideration and assess the comprehensive impact of LAP and nutritional factors on the onset of diabetes. Fifth, the current study only assessed the predictive significance of baseline LAP for future diabetes in both sexes; it is hoped that the longitudinal effects of LAP increase/decrease on blood glucose metabolism (including progression to diabetes and return to normal blood glucose metabolism) in both sexes can be further evaluated in the future, which is of great value for precision therapy.

Despite these limitations, the NAGALA cohort’s large representative sample size, prospective study design with longer continuous follow-up, detailed and standardized assessment of multiple demographics, laboratory test data and lifestyle, construction of multivariate regression models to adjust for multiple potential confounders, and use of ROC analysis incorporating time factors combined to provide robust support for the current findings, which together led us to prefer LAP as the more appropriate obesity parameter for females. As far as we know, the above results based on time-dependent ROC analysis are all reported for the first time.

## 5 Conclusion

In conclusion, our study provides robust support for the stronger association between LAP and diabetes risk in females compared to males. The predictive accuracy and stability of LAP for diabetes were also higher in females with fewer fluctuations in the threshold values. These findings underscore the importance of considering gender disparities when assessing the relationship between visceral fat and future diabetes risk. In particular, our findings suggested that gender disparities in LAP were of great relevance for predicting diabetes, highlighting the potential for developing sex-specific and individualized strategies for diabetes prevention and control. Overall, these findings emphasize the need for healthcare workers and public health professionals to consider the impact of gender differences when assessing the risk of diabetes.

## Data availability statement

The datasets presented in this study can be found in online repositories. The names of the repository/repositories and accession number(s) can be found in the article/**Supplementary Material**.

## Ethics statement

The studies involving humans were approved by the Ethics Committee of Jiangxi Provincial People’s Hospital. The studies were conducted in accordance with the local legislation and institutional requirements. The ethics committee/institutional review board waived the requirement of written informed consent for participation from the participants or the participants’ legal guardians/next of kin because According to local laws and regulations, the Ethics Committee of Jiangxi Provincial People’s Hospital exempted the repeated signing of informed consent of subjects (IRB:2021-066).

## Author contributions

JQ: Data curation, Software, Writing – original draft. MK: Formal Analysis, Software, Visualization, Writing – original draft. YZ: Software, Writing – original draft, Writing – review & editing. RY: Data curation, Formal Analysis, Visualization, Writing – review & editing. QS: Formal Analysis, Visualization, Writing – review & editing. DL: Formal Analysis, Visualization, Writing – review & editing. GS: Conceptualization, Data curation, Supervision, Writing – review & editing. WW: Conceptualization, Data curation, Supervision, Writing – review & editing.
