# Supplementary figures and images for: The predictive significance of lipid accumulation products for future diabetes in a non-diabetic population from a gender perspective: an analysis using time-dependent receiver operating characteristics
Source: Front Endocrinol (Lausanne). 2023 Nov 14;14:1285637. doi: 10.3389/fendo.2023.1285637 (PMC10682705; doi:10.3389/fendo.2023.1285637)

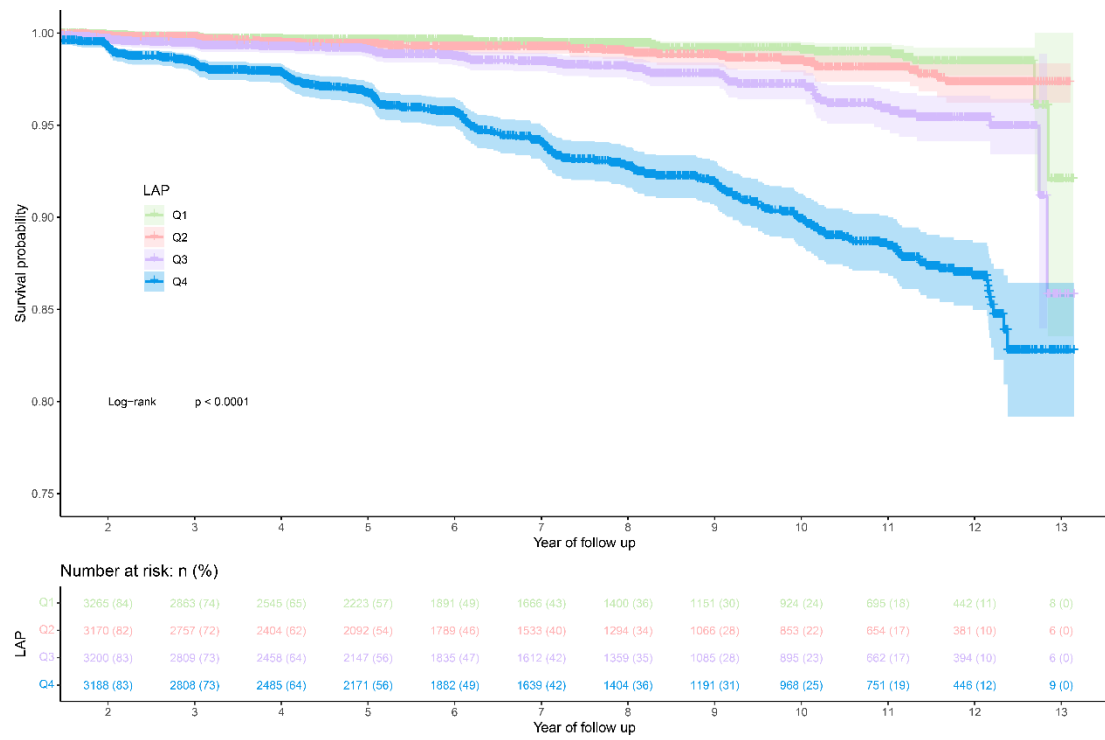

**Supplementary Figure 1: Kaplan-meier curve of LAP quartiles over time.**

Supplement: Supplementary file 1 [file Image_1.pdf]
