# Supplementary material for: The predictive significance of lipid accumulation products for future diabetes in a non-diabetic population from a gender perspective: an analysis using time-dependent receiver operating characteristics
Source: Front Endocrinol (Lausanne). 2023 Nov 14;14:1285637. doi: 10.3389/fendo.2023.1285637 (PMC10682705; doi:10.3389/fendo.2023.1285637)
Supplement: Supplementary file 2 [file Table_1.docx]

Supplementary Table 1: Collinearity diagnostics steps of LAP with other covariates.

|  | VIF | | | | |  |
| --- | --- | --- | --- | --- | --- | --- |
|  | **Step 1** | **Step 2** | **Step 3** | **Step 4** | **Step 5** | |
| LAP | 12.5 | 12.1 | 8.5 | 2.3 | 2.3 | |
| Sex | 3.7 | 3.7 | 3.6 | 3.2 | 3.2 | |
| Age | 1.4 | 1.4 | 1.3 | 1.3 | 1.3 | |
| Height | 54 | 2.8 | 2.6 | 2.4 | 2.4 | |
| Weight | 175.7 | NA | NA | NA | NA | |
| BMI | 99 | 5.1 | 3 | 2.1 | 2.1 | |
| WC | 8.6 | 8.5 | NA | NA | NA | |
| ALT | 4.1 | 4.1 | 4.1 | 4.1 | 4.1 | |
| AST | 3.3 | 3.3 | 3.3 | 3.3 | 3.3 | |
| GGT | 1.5 | 1.5 | 1.5 | 1.5 | 1.5 | |
| HDL-C | 1.8 | 1.8 | 1.8 | 1.7 | 1.7 | |
| TC | 1.5 | 1.5 | 1.4 | 1.4 | 1.4 | |
| TG | 8.5 | 8.3 | 6.4 | NA | NA | |
| HbA1c | 1.1 | 1.1 | 1.1 | 1.1 | 1.1 | |
| SBP | 5.6 | 5.6 | 5.6 | 5.6 | 1.4 | |
| DBP | 5.7 | 5.7 | 5.7 | 5.7 | NA | |
| Fatty liver | 1.6 | 1.6 | 1.6 | 1.6 | 1.6 | |
| Exercise habits | 1 | 1 | 1 | 1 | 1 | |
| Drinking status | 1.3 | 1.3 | 1.3 | 1.3 | 1.3 | |
| Smoking status | 1.4 | 1.4 | 1.4 | 1.4 | 1.4 | |

Abbreviations: VIF: Variance inflation factor; Other abbreviations as in Table ​1.

Note: VIF = 1/(1-R^2^).
